# Supplementary material for: Short-Term Exposure to High Atmospheric Vapor Pressure Deficit (VPD) Severely Impacts Durum Wheat Carbon and Nitrogen Metabolism in the Absence of Edaphic Water Stress
Source: Plants (Basel). 2021 Jan 8;10(1):120. doi: 10.3390/plants10010120 (PMC7827516; doi:10.3390/plants10010120)
Supplement: Supplementary file 1 [file plants-10-00120-s001.pdf]

Table S1. Sequences of the primers used to amplify the cDNA of the RuBisCO, nitrogen transport and assimilation genes.

Gene	Acc. No.	Forward	Reverse
TaRBCL	EU492898	CATTCCGAGTAAGTCCTCAGC	CAGTAGAAGATTCGGCAGCT
TaRcaB	AF251264	GGGATGTACAACAAGGAGGAG	CCCAGTAGAACTTCTCCATGC
TaNRT1.3	HF544990	CACCTGCATCCTCTTCTGG	AGGATGGAGAGGAAGAGGAAG
TaNRT1.4	HF544991	CTTACCTCCTATGCGCCATG	ACCAGCTAGCATTTCTCGTC
TaNRT1.5	HF544992	GCAACTACTTCAGCGACATC	GAGAGTATGGCTAGCAGGAAG
TaNRT1.7	HF544995	TCCTTTATCGCCCTAACTGC	CACCAATCCTATACCCTGTCTC
TaNRT1.8	HF544998	CTCGAGATAAAGAGGCTGGC	GGAAGTACTGAGGGATTTGCC
TaNRT1.9	HF544999	CCGTGAACCTCGTCTACTTTG	GTCATCGAGCCCAACATCTC
TaGS2	DQ124212	GTACATCTGGGTTGGAGGATC	GGTGCTCGATCCGTCATAAT
TaNIR	FJ527909	AACCTCCTCTCCTCCTACATC	TACGCCAGGTCGTTGATATG
TaActin	AB181991	AGAGTCGGTGAAGGGGACTTA	TCCTGTACCCCTTATTCCTCTGA

Table S2. P and F values of the different parameters analysed Figures 2–4, based on one-way ANOVA. Bold values indicate statistical difference ($p < 0.05$).

Parameter	F-value	P-value
A_N	17.32	0.001
E	11.51	0.004
g_s	7.27	0.015
C_i	0.77	0.54
V_{Cmax}	1.42	0.33
J_{max}	4.26	0.06
V_{Cmax}/J_{max}	1.62	0.28
$\delta^{18}O$	15.62	0.001
$\delta^{15}N$ non-labelled	2.09	0.179
$\delta^{15}N$ labelled	12.77	0.002
$\delta^{13}C$-TOM	3.19	0.084
$\delta^{13}C$-WSC	29.09	0.000
RBCL	6.27	0.003
RcaB	5.58	0.005
NRT1.3	2.12	0.125
NRT1.5	0.38	0.766
NRT1.8	9.74	0.000
NRT1.4	8.83	0.000
NRT1.7	0.21	0.890
NRT1.9	1.61	0.214
GS2	7.68	0.001
NIR	2.40	0.094

Table S3. P and F values of the different parameters analysed in Table 1, based on one-way ANOVA. Values in bold indicate statistical significance ($p < 0.05$).

Parameter	F-value	P-value
ARG	21.52	0.000
LYS	16.48	0.001
LEU	14.90	0.001
ILE	15.63	0.001
MET	11.48	0.003
PHE	11.77	0.003
TRP	9.18	0.005
HIS	3.77	0.06
TYP	21.58	0.000
VAL	11.16	0.003
GLN	13.49	0.002
PRO	6.10	0.018
ASN	0.96	0.460
GABA	0.87	0.495
THR	3.68	0.062
SER	3.99	0.052
GLY	3.55	0.067
ALA	2.87	0.103
GLU	0.44	0.73
ASP	2.58	0.126
